# Clinical verification of genetic results returned to research participants: findings from a Colon Cancer Family Registry

**DOI:** 10.1002/mgg3.328

**Published:** 2017-08-23

**Authors:** Mercy Y. Laurino, Anjali R. Truitt, Lederle Tenney, Douglass Fisher, Noralane M. Lindor, David Veenstra, Gail P. Jarvik, Polly A. Newcomb, Stephanie M. Fullerton

**Affiliations:** ^1^ Cancer Prevention Program Seattle Cancer Care Alliance Seattle Washington USA; ^2^ Department of Rehabilitation Medicine University of Washington Seattle Washington USA; ^3^ Public Health Sciences Division Fred Hutchinson Cancer Research Center Seattle Washington USA; ^4^ Department of Health Sciences Research Mayo Clinic Scottsdale Arizona USA; ^5^ Pharmaceutical Outcomes Research and Policy Program School of Pharmacy University of Washington Seattle Washington USA; ^6^ Division of Medical Genetics Department of Genome Sciences University of Washington Seattle Washington USA; ^7^ Department of Bioethics and Humanities University of Washington Seattle Washington USA

**Keywords:** Cancer registry, CLIA verification, genetic research, Lynch syndrome, return of research results

## Abstract

**Background:**

The extent to which participants act to clinically verify research results is largely unknown. This study examined whether participants who received Lynch syndrome (LS)‐related findings pursued researchers’ recommendation to clinically verify results with testing performed by a CLIA‐certified laboratory.

**Methods:**

The Fred Hutchinson Cancer Research Center site of the multinational Colon Cancer Family Registry offered non‐CLIA individual genetic research results to select registry participants (cases and their enrolled relatives) from 2011 to 2013. Participants who elected to receive results were counseled on the importance of verifying results at a CLIA‐certified laboratory. Twenty‐six (76.5%) of the 34 participants who received genetic results completed 2‐ and 12‐month postdisclosure surveys; 42.3% of these (11/26) participated in a semistructured follow‐up interview.

**Results:**

Within 12 months of result disclosure, only 4 (15.4%) of 26 participants reported having verified their results in a CLIA‐certified laboratory; of these four cases, all research and clinical results were concordant. Reasons for pursuing clinical verification included acting on the recommendation of the research team and informing future clinical care. Those who did not verify results cited lack of insurance coverage and limited perceived personal benefit of clinical verification as reasons for inaction.

**Conclusion:**

These findings suggest researchers will need to address barriers to seeking clinical verification in order to ensure that the intended benefits of returning genetic research results are realized.

## Introduction

Notwithstanding emerging ethical consensus that it is desirable to return clinically actionable results from genetic research to participants (Fabsitz et al. 2010; Jarvik et al. 2014; Knoppers and Laberge 2009), controversy persists with regard to whether or not such results ought to be verified in a CLIA‐certified laboratory prior to return (Bookman et al. 2006; Jarvik et al. 2014; Wolf et al. 2008, 2012). While some argue that non‐CLIA‐verified results should never be returned (Dressler 2009), a CLIA research exception does exist, which allows individual genetic results to be returned with the caveat that such results are verified by a CLIA‐certified laboratory before clinical action is taken (Burke et al. 2014; Evans 2014). Furthermore, Evans has suggested that the return of non‐CLIA results to willing participants may be permissible as a First Amendment right (Evans 2014).

To date, no study has documented the extent to which participants pursue CLIA verification of genetic results received in a cancer research setting. In 2013, Siegfried et al. described 23 individuals in 10 families with Familial Dilated Cardiomyopathy who verified research results in CLIA‐certified laboratories but did not report how many participants failed to clinically verify their results or explore perceived barriers associated with the pursuit of clinical verification (Siegfried et al. 2013). If the primary ethical rationale for result return is beneficence, it is important to consider whether participants pursue such verification when it is recommended by the research team. To explore this question, we conducted a study of postresult disclosure clinical (CLIA‐certified) result verification as reported by participants in the Fred Hutchinson Cancer Research Center (FHCRC) Colon Cancer Family Registry (Seattle C‐CFR).

The Seattle C‐CFR is one of six sites in a multinational cancer registry which has pursued research‐related genetic testing using biospecimens donated by participants (Newcomb et al. 2007). After local institution review board (IRB) approval, the Seattle C‐CFR began offering genetic results to participants in February 2011 (Keogh et al. 2014). Participants selected for result return belonged to families identified as having a segregating Lynch syndrome (LS) (OMIM #120435)‐related pathogenic variant in one of four mismatch repair (MMR) genes (i.e., *MLH1*,* MSH2*,* MSH6*, and *PMS2* [MIM*120436, MIM*609309, MIM*600678, MIM*600259, respectively], Berg et al. 2009; Clayton and Ross 2006; Palomaki et al. 2009; Weissman et al. 2012). Briefly, LS, also known as hereditary nonpolyposis colorectal cancer (HNPCC), is a hereditary colon cancer syndrome that increases an affected individual's risk for cancers of the colon, rectum, stomach, hepatobiliary tract, endometrium, and ovaries (Hampel et al. 2008; Kohlmann and Gruber 2004; Win et al. 2012, 2013). The Seattle C‐CFR's decision to offer these findings to participants was based on the high predictive value of the identified MMR pathogenic variants and the accepted clinical utility of LS genetic testing.

## Materials and Methods

### Ethical compliance

This study was approved by the University of Washington and Fred Hutchinson Cancer Research Center's Institutional Review Boards (C‐CFR consortium project # C‐EX‐0806‐05).

### GenBank accession numbers

For the genes in this study, the GenBank accession numbers were: *MLH1* NM_000249.3, *MSH2* NM_000251.2, *MSH6* NM_00179.2, *PMS2* NM_000535.5.

### Result disclosure protocol

Using established protocols, Seattle C‐CFR participants for whom genetic research testing identified a pathogenic variant in the *MLH1*,* MSH2*,* MSH6*, or *PMS2* genes, and their enrolled family members who were presumptively tested for the familial variant, were contacted for an opportunity to opt‐in for result return from the registry (Keogh et al. 2014). Following informed consent, interested participants were asked to attend two genetic counseling appointments: the first session was designed to discuss issues associated with learning genetic results from the registry; participants were then asked to attend a second genetic counseling session where results were returned. Midway through the return process, however, the study team received feedback that participants preferred to receive their results at the end of the first appointment. The protocol was therefore modified such that 23 (67.6%) of the participants received results during a second genetic counseling session, whereas 11 (32.4%) received results at the end of a single genetic counseling session. Due to the small sample sizes, we choose not to stratify respondents by their return protocol.

The participants who elected to receive results were told that their findings were preliminary, that is, for information purposes only, and counseled on the importance of verifying their results in a CLIA‐certified laboratory; all provided informed consent of this understanding. Participants who received counseling and research results were sent a follow‐up summary letter, as well as a letter addressed to a health care provider identified by the participant. It was the participant's responsibility to share the letter with his or her provider. In both letters, the recommendation to verify the result in a CLIA‐certified laboratory was emphasized. Upon request, the study's genetic counselor (ML) provided contact information of clinical genetic counseling services. Participants who elected to receive results were invited to participate in research follow‐up surveys and interviews designed to explore whether and how they had acted on the information received. The decision to participate in these surveys did not affect the opportunity to receive research results.

### Postdisclosure assessment

A sequential mixed‐method design was employed to examine self‐reported postdisclosure attitudes and actions.

All participants who received research results were first invited to participate in postdisclosure surveys that included questions focused on (1) verification of results by a CLIA‐certified laboratory and (2) family and health care provider communication (the latter data are not presented here). From April 2011 to April 2014, the surveys were conducted by the study interviewer (LT) over the phone. Participants were called up to three times at each time point (2 and 12 months) to maximize survey participation.

We then invited a subset of the participants to participate in a semistructured follow‐up interview. The aim of these interviews, completed at least 12 months postdisclosure, was to explore with participants their reasons for pursuing, or not pursuing, clinical verification of their results at a CLIA‐certified laboratory. A draft interview guide was first reviewed and refined by the study team to ensure clarity and conversational tone; the guide was not formally piloted, however, due to the small number of potential participants. Interview questions included: *How long ago were you contacted to learn of research results from your participation in the registry? What was your experience when you were initially contacted? Can you please describe how you came to decide that you wanted to know (or not know) your genetic research results? As recommended when you spoke with the genetic counselor, did you have your research results confirmed in a clinical laboratory? If yes, what was the process like? If no, why did you decide not to pursue confirmation?* Participant interviews were conducted between April 2014 and October 2014, lasting on average of 19 min. Interviews were conducted over telephone, audio‐recorded, and transcribed for qualitative analysis.

### Data analysis

#### Quantitative analyses

Postdisclosure survey responses and demographic information (age, gender, education, marital status, educational background, and race) were collected by telephone, manually added to a database, and cross‐checked to ensure accuracy. Survey data were subsequently deidentified, downloaded for analysis with STATA14 statistical software (2015). Descriptive characteristics (e.g., participant characteristics, survey completion status, interview status, and reports of verification of results at CLIA‐certified laboratories) were summarized, including frequencies, means, and standard deviations.

#### Qualitative analyses

Follow‐up interviews were transcribed verbatim. Transcripts were checked for accuracy and uploaded to Atlas.ti (1999) for analysis (Krippendorff 2013). A preliminary code list was created based on the topics discussed during the interview, and additional codes were added based on the themes shared by the participants. Two research team members (ML, AT) independently coded the transcripts for validity. By constant comparison (Seale 2007) of the generated codes, the content analysis offered a systematic approach to identifying the experiences of the Seattle C‐CFR participants postresult disclosure.

## Results

### Seattle C‐CFR return of results

**Figure 1:**
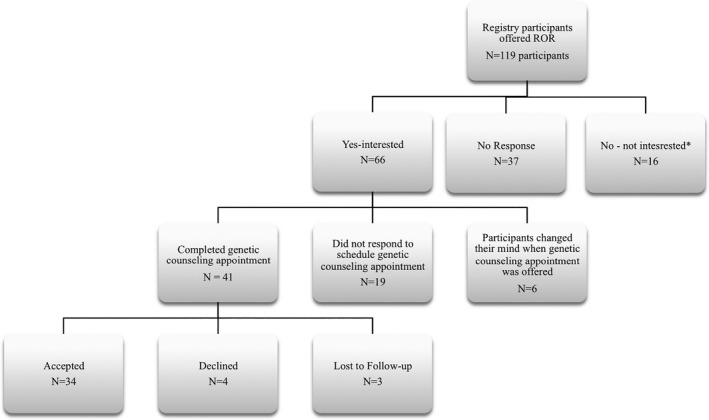
Seattle C‐CFR return of results (ROR) process (February 2011–April 2013).

Of approximately 8000 total (Colon Cancer Family Registry [Link]) Seattle C‐CFR participants, 119 met inclusion criteria for recontact and were offered the opportunity to learn LS‐related genetic research findings (Fig. 1). Criteria for inclusion was identification as a case (i.e., affected with CRC and the first person in the family enrolled in the registry) with genetic testing that revealed a LS‐related pathogenic variant, or an enrolled family member who had been tested for the same pathogenic variant (irrespective of test result). Among the participants approached for result return from February 2011 to April 2013, 55.5% (66/119) expressed an interest in learning their results. Of these, 62.1% (41/66) completed their genetic counseling sessions, and 82.9% (34/41) of those elected to receive their research results. Of the 34 participants who accepted results, 26 (76.5%) completed both the 2‐ and 12‐month posttest disclosure surveys. The majority of the surveys were completed by women: 60.9% (14/23) at 2 months and 57.7% (15/26) at 12 months. Eleven of 26 participants invited for the semistructured interview chose to participate, for a final response rate of 42.3%. Characteristics of participants who received results, completed surveys, and participated in interviews are shown in Table 1.

**Table 1:** Characteristics of participants who completed posttest disclosure survey and follow‐up interview

	Completed 2‐month^a^	Completed 12‐month^b^	Participated in interview
	*N* (%)	*N* (%)	*N* (%)
Age, years	48.8±14.5; 20–73	48±13.6; 20–73	47.9±10.9; 36–72
Sex			
Male	9 (39.1)	11 (42.3)	1 (9.1)
Female	14 (60.9)	15 (57.7)	10 (90.9)
Had children			
No	8 (34.8)	8 (30.8)	4 (36.4)
Yes	15 (65.2)	18 (69.2)	7 (63.6)
Personal history of cancer			
Colon	6 (26.1)	6 (23.1)	4 (36.4)
Other cancers^c^	2 (8.7)	2 (7.7)	3 (27.3)
None	15 (65.2)	18 (69.2)	4 (36.4)
Number of family members with cancer			
≤1	5 (21.7)	5 (19.3)	1 (9.1)
2–3	18 (78.3)	21 (80.8)	10 (90.9)
Education level			
Less than college	6 (26.1)	8 (30.8)	3 (27.3)
Some college	10 (43.5)	10 (38.5)	3 (27.3)
College graduate	7 (30.4)	8 (30.8)	5 (45.5)
Unknown	–	–	–
Race			
Caucasian	15 (65.2)	17 (26.9)	10 (90.9)
Non‐Caucasian	2 (8.7)	2 (7.7)	1 (9.1)
Unknown	6 (26.1)	7 (26.9)	–
Participant^d^			
CRC case	4 (17.4)	4 (15.4)	4 (36.4)
Affected relative	2 (8.7)	3 (11.5)	3 (27.3)
Unaffected relative	16 (69.6)	19 (73.1)	4 (36.4)
MMR gene status^e^			
Positive for pathogenic variant	13 (56.5)	14 (53.8)	10 (90.9)
Negative for pathogenic variant	10 (43.5)	12 (46.2)	1 (9.1)

a One participant completed the 2‐month but not the 12‐month posttest disclosure survey.

b Four participants completed the 12‐month but not the 2‐month posttest disclosure survey.

c Participants diagnosed with biliary/renal, ovarian, stomach, pancreas, bile duct, or small bowel cancers.

d Case = participant with colon cancer and first enrollee of the family; relative = enrolled family member.

e MMR gene and GenBank accession numbers are: *MLH1* NM_000249.3, *MSH2* NM_000251.2, *MSH6* NM_00179.2, *PMS2* NM_000535.5.

### CLIA verification of research findings: survey results

Within 12 months of result disclosure, 15.4% (4/26) of participants surveyed reported having verified results in a CLIA‐certified laboratory. Interestingly, whereas only 15 (57.7%) of the 26 who completed the 12‐month survey were women, all who reported clinically verifying their research results were women. Three (75%) of the 4 participants in this category received results characterized as disease‐associated MMR pathogenic variants and one had tested negative for the familial mutation; and research and clinical results were in each case concordant.

Of those who did not report verifying their results in a CLIA‐certified laboratory, 2 (7.7%) of 26 planned to verify but had not done so by 12 months postresult disclosure, 3 (11.5%) did not verify because the clinical test cost was not covered by their insurance, 3 (11.5%) did not have insurance coverage, and 1 (3.8%; 1/26) reported not verifying because they did not want their insurance company to know their status. There were 8 (30.8%; 8/26) participants who said that they did not feel the need to repeat their test results. Of these, 4 (50%; 4/8) had positive LS‐related results and 2 (50%; 2/4) belonged to families in which another family member had clinically verified their results. Five participants (19.2%; 5/26) did not specify why they chose not to pursue clinical verification of their findings.

### CLIA verification decision making: findings from interviews

Eleven participants (4 cases [36.4%] and 7 relatives [63.6%]) who received results and completed postdisclosure surveys were subsequently interviewed (Table 1). Of these, 10 (90.9%) tested positive for a pathogenic variant in one of the LS‐related MMR genes, and 4 (36.4%) reported verifying their research results at a CLIA‐certified laboratory.

#### Reasons for clinical verification

The four participants who reported clinically verifying their research results gave two major reasons for choosing to verify at a CLIA‐certified laboratory: (1) acting on the recommendation of the research team, and (2) informing future clinical care.

Most scheduled their consultations at genetic services as recommended by the Seattle C‐CFR genetic counselor, sometimes with the direct help of the study staff. “She [the genetic counselor] said that we need to test you just to verify that you, in fact, carry the gene [sic]. So right there, they sent me to the lab and had blood work done, and I just confirmed that I carry the gene” (P6, participant with LS).

Another important rationale for verifying research results was the recognition of the clinical utility of findings, which might help health care providers make recommendations about appropriate cancer screening surveillance and prophylactic surgery. For example, one participant had already pursued a hysterectomy for cancer prevention but nevertheless saw the information as being useful for other surgical decision making. “I don't have my uterus already because I had a hysterectomy prior to knowing this, but I still have my ovaries, so they also recommended that I [clinically confirm] to remove the ovaries and fallopian tube” (P8, participant with LS).

All of the interviewed participants reported pursuing the recommended cancer screening and surveillance in light of their genetic test findings.

#### Reasons to not clinically verify

For those participants who reported not having verified their research findings in a CLIA‐certified laboratory by the time of the interview (completed at least 12 months postdisclosure), two major reasons were noted: (1) lack of insurance coverage or other insurance‐related concerns, and (2) limited perceived additional personal or clinical benefit.

Two participants reported that lack of insurance or limited funding had prevented them from pursuing the recommendation to verify their results at CLIA‐certified laboratories. “I can't. I checked with a doctor, but if it's going to cost me, then no, but otherwise I'll just go with what I know and so next time I'll go to the doctor I'll check to see what she thinks and take it from there” (P9, participant with LS). However, both indicated that they understood the importance of clinical verification, and stated that they planned to follow‐up with their health care provider soon.

Two other participants reported feeling that they did not need to verify the research results as they already had colon cancer. “Because I didn't see any reason to … I already had cancer figured out. I got the syndrome, what's the big deal?” (P4, participant with LS) These participants also knew of enrolled family members who had elected to receive their results from the registry and who had subsequently verified those findings. “I didn't. No, [but] my sister did. I experienced having colon cancer at a young age. I just felt that the study was adequate enough” (P3, participant with LS).

## Discussion

Despite concerted efforts to ensure participant understanding about the importance of clinical verification, our results suggest that only a minority (15.4%; 4/26) of the Seattle C‐CFR participants who received LS‐related findings chose to verify those findings in a CLIA‐certified laboratory within 12 months of receiving their results. For those who participated in qualitative interviews, participants who clinically verified their results shared that acting on the recommendation of the research team and informing future clinical care were the main reasons they pursued verification at a CLIA‐certified laboratory. Those participants who did not clinically verify results explained that their lack of insurance coverage or the limited perceived personal and/or clinical benefits were reasons for not acting on the study team's recommendation. Some participants reported intending to pursue clinical verification of results but experiencing personal barriers (e.g., limited funds, insurance coverage, etc.) that prevented them from doing so. To our knowledge, this is the first study to provide empirical data on whether or not participants in a cancer registry pursue non‐CLIA research result verification in CLIA‐certified laboratories. Although Graves et al. published a description of the Mayo C‐CFR's result return effort, that report did not examine whether participants clinically verified their research findings (Graves et al. 2013).

Clinical Laboratory Improvement Amendments of 1988 (CLIA) requires that laboratories meet quality standards to ensure patients receive accurate, reliable, and timely results. Laboratory test results used in clinical care must therefore be performed in CLIA‐certified laboratories while tests performed in research laboratories are not held to the same quality standards. A CLIA research exception explicitly states that results of tests performed in non‐CLIA‐certified laboratories should be used for informational purposes only (Burke et al. 2014; Evans 2014), and with the expectation that participants will subsequently clinically verify their results. However, some Seattle C‐CFR participants reported feeling that they did not need to clinically confirm their research results given their personal history of cancer and/or because they knew of other enrolled family members who had received and clinically verified their research findings. We do not know whether these participants sought out clinical treatment (e.g., enhanced surveillance, prophylactic surgery) despite unverified results. In general, health care providers are involved whenever participants take any clinical actions (e.g., a woman with LS seeking a hysterectomy for cancer risk reduction) and it is unlikely that a health care provider would make recommendations based solely on a genetic result generated by a non‐CLIA‐certified laboratory. Of course, it is possible that a patient's research result could be utilized within the context of the participant's medical and family history of cancer, as well as knowing a family members’ pursuit of clinical verification of their own research findings. In such cases, even unverified information of the research result at a CLIA‐certified laboratory could be valuable.

While newer genetic research studies typically plan for collecting and processing samples in a CLIA‐compliant fashion when result return is anticipated, analyses of older samples from ongoing studies are often not CLIA‐compliant. In such cases, return of non‐CLIA results to a willing participant may be the only viable option for conveying research findings of potential health relevance (Burke et al. 2014; Jarvik et al. 2014). Nevertheless, controversy continues whether or not individual genetic research results ought to be verified in a CLIA‐certified laboratory prior to return (Bookman et al. 2006; Jarvik et al. 2014; Wolf et al. 2008, 2012). In a recent analysis, Evans argued that the communication of research results generated from non‐CLIA‐certified laboratories to willing participants may be regarded as a speech act protected by the First Amendment (Evans 2014). Moving forward, if verification of research results in CLIA‐certified laboratories becomes the default expectation for result return efforts, researchers will need to obtain additional resources from funding agencies to support it.

Routine CLIA verification of research findings may, however, pose other concerns. Burke and colleagues recently emphasized that the research result return process “…should be focused on clarity, appropriate caveats and, most important, appropriate referrals when the results may be helpful to consider in clinical care” in order to minimize the risk of therapeutic misconception (Burke et al. 2014). In other words, participants may mistakenly believe that the main purpose of their participation in research is to provide them with medical benefit rather than advancing knowledge when researchers return clinically actionable research findings (Burke et al. 2014; Clayton and Ross 2006; Resnik 2011; Wolf et al. 2015). In one cancer research study in Ontario, Canada, for example, Miller and colleagues showed that result return made research participation seemed more like receiving “quasi‐clinical” care from the research team (Miller et al. 2008). As Clayton and McGuire (2012) note, an emerging consensus for return of research results to become “standard of care” may impose an unwarranted legal (negligence‐based) duty on investigators.

### Study limitations

The Seattle C‐CFR returned research results to only a small number of registry participants and, accordingly, we were only able to collect survey responses from, or conduct follow‐up interviews with, small numbers of respondents. This limited our ability to draw statistically significant inferences or to ensure that we reached saturation with our qualitative observations. We were also unable to interview participants who chose not to pursue clinical genetic testing or who declined the result return opportunity. Furthermore, interview findings were likely influenced by recall bias as the participants were interviewed at least a year after they received their research results. Finally, the responses we received were possibly influenced by underlying participant characteristics and we tried to minimize this bias by offering research results to all eligible participants who met the inclusion criteria.

Overall, with the Seattle C‐CFR return of research results protocol, care was taken to distinguish research from clinical care by providing each participant and their provider with information on clinical services that would assist them with verification of the research results. Participants also provided informed consent of their understanding that they were receiving research results obtained from a non‐CLIA‐certified laboratory and the importance of clinical verification. Nevertheless, some participants still shared that they did not intend to verify their results given their own perceived lack of personal utility. In addition, with or without clinical verification, Seattle C‐CFR participants reported sharing their research results with their family members (data not shown). While this outcome supports current public health efforts to identify individuals with LS in an effort to prevent cancer in at‐risk relatives (Bellcross et al. 2011; Berg et al. 2009; Hampel and de la Chapelle 2011; Hampel 2014), this issue will need further investigation in order to ensure that clinical verified genetic information forms the basis for familial communication about disease risk.

## Conclusion

Only a minority of Seattle C‐CFR participants in this study acted to clinically verify non‐CLIA LS‐related findings. This result suggests that a variety of factors may prevent participants from pursuing routine research result verification performed at CLIA‐certified laboratories. As such, researchers will need to consider the potential for such barriers in their own studies. It is important to ensure that participants both understand the advantages of non‐CLIA research result verification and that they have access to the appropriate resources for clinical testing.

## Conflict of Interest

There are no conflicts of interest.
